# Detection of novel *Wolbachia* strains in *Aedes aegypti* populations from a recent arbovirus outbreak region in Pune District, Maharashtra, India (2024)

**DOI:** 10.3389/fmicb.2026.1766962

**Published:** 2026-02-25

**Authors:** Irrusappan Hari, Tharani Priya Panner Selvam, Sanket Kumar Ray, Alagarasu Kalichamy, Vikas Sharma, Kannan Thiruvengadam, Kavita Satish Lole, Ashwini Ramdasi, Supriya Hundekar, Pranit Vijay Ayachit, Prajwal Gaikwad, Balasubramanian Rathinam, Somaji Shankar Anuse, Kalpana Baruah

**Affiliations:** 1 ICMR-National Institute of Virology, Pune, India; 2 Academy of Scientific and Innovative Research, Ghaziabad, Uttar Pradesh, India; 3 ICMR-Vector Control Research Centre, Puducherry, India; 4 Bharathidasan University, Tiruchirappalli, Tamil Nadu, India; 5 Public Health Department, State Government of Maharasthtra, India; 6 National Center for Vector Borne Diseases Control, Delhi, India

**Keywords:** *Aedes*, chikungunya, dengue virus, novel strain, vector control, *Wolbachia*

## Abstract

*Aedes aegypti* mosquitoes are principal vectors of arboviruses such as dengue, chikungunya, and Zika. The intracellular symbiont *Wolbachia pipientis* is known to inhibit viral replication and induce cytoplasmic incompatibility, making it a promising candidate for biological vector control. While *Wolbachia* is commonly found in *Ae. albopictus*, its natural presence in *Ae. aegypti* remains under debate, particularly in India. This study investigated the presence and diversity of *Wolbachia* in *Ae. aegypti* mosquitoes collected from 21 locations across Pune district, Maharashtra, during a 2024 arbovirus outbreak. A total of 1,020 adult mosquitoes and 1,000 larvae and pupae were morphologically and molecularly confirmed as *Ae. aegypti* and pooled (*n* = 93) for *Wolbachia* screening using 16S rRNA and *wsp* gene-specific PCRs. Positive samples were sequenced and subjected to phylogenetic and intergenomic similarity analyses. Simultaneously, dengue, chikungunya, and Zika virus screening was conducted via RT-qPCR. Entomological indices were calculated to assess vector density. *Wolbachia* was detected in 11.8% of *Aedes aegypti* pools. Phylogenetic and similarity analyses identified three distinct clusters: supergroup A (*n* = 2), B (*n* = 5), and a divergent F-type strain (*n* = 2). Nucleotide gene sequence similarity analysis corroborated the phylogenetic structure, showing high intra-supergroup similarity and low inter-supergroup similarity, consistent with deep evolutionary divergence among supergroups. Supergroup A and B sequences exhibited close affinity to known *wAlbA* and *wAlbB* lineages, respectively, whereas the F-type sequence formed a distinct cluster with low intergenomic similarity to A and B members, indicating a divergent lineage. Dengue virus RNA was detected in two pools, one co-occurring with *Wolbachia*, although individual co-infection could not be confirmed. This study provides the first evidence of naturally occurring *Wolbachia* supergroups A, B, and a potentially novel F-type in *Ae. aegypti* from an arbovirus-endemic region of western India. These findings highlight the evolutionary diversity of *Wolbachia* in local vector populations and underscore the importance of integrating *Wolbachia* surveillance into vector control strategies.

## 1 Introduction

Vector-borne diseases remain an important health threat across the globe, with mosquito vectors belonging to the family *Culicidae* playing crucial roles in spreading lethal pathogens (Onen et al., 2023; Nebbak et al., 2022; Socha et al., 2022; Mayer et al., 2017). Arthropod-borne viruses (arboviruses) are defined as viruses transmitted by arthropods such as mosquitoes, ticks, and sandflies. Among them, dengue virus (DENV), chikungunya virus (CHIKV), and Zika virus (ZIKV) are primarily transmitted by mosquitoes, particularly *Aedes* species (Nebbak et al., 2022; Mayer et al., 2017; Artsob et al., 2025; Huang et al., 2019; Socha et al., 2022; Somia et al., 2023). Among the vector-borne diseases (VBD), arboviral diseases alone account for 17% of infectious diseases and around 700,000 deaths per year worldwide (Ahmed et al., 2019; Tajudeen et al., 2021; WHO, 2025). Among the arboviruses, DENV alone is known to infect an estimated 390 million individuals in 128 endemic countries (Ahmed et al., 2019; Tejo et al., 2024). These infections are transmitted primarily by *Ae. aegypti* mosquitoes, which thrive in urban and semi-urban environments and breed in artificial water containers near human dwellings (Abbasi, 2025; Bikangui et al., 2023; Jangir and Prasad, 2022; Padonou et al., 2023; WHO, 2016). India, with a population size of approximately 1.45 billion (as of 2024), is the world’s most populous country. Rapid urbanization, population density, and favorable climatic conditions facilitate the spread of disease vectors across the country (Naik et al., 2023).

Dengue outbreaks have significantly increased over the years in India, with chikungunya and Zika epidemics being reported in certain areas (Soni et al., 2023; Soni et al., 2023; Dinesh et al., 2025; Nayak et al., 2025). Pune is a significant Metropolitan city located in the state of Maharashtra in India with a constant growth in population. The city, as of 2025, depicts an estimated annual population growth of about 179,900 people (WPR, 2025). The city has experienced a significant arbovirus outbreak in recent years (Arankalle et al., 2024; Alagarasu et al., 2023a; Alagarasu et al., 2023b; Gurav et al., 2022). Vector control is necessary to prevent the spread of vector-borne diseases and reduce the burden caused by outbreaks of illnesses like dengue, Zika, and chikungunya, and other arboviral diseases that often lack effective antiviral therapies or vaccines (Paixão et al., 2018; Shaw and Catteruccia, 2018; Montenegro et al., 2024; Zhang et al., 2024). Vector control involves chemical, biological, mechanical, and environmental interventions designed to kill the vectors or limit their capacity to reproduce, bite, or transmit disease (Bouzid et al., 2016; Huang et al., 2017; Rafikov et al., 2015). In such vector control measures, chemical vector control aims to reduce the mosquito population through the use of insecticides. Although this approach demonstrates successful practical application against the *Aedes* mosquito, the challenges including the development of insecticide resistance, possible ecological implications, and the limited scope of application, are some factors that restrict its long-term sustainability. The *Ae. aegypti* population in India is increasingly resistant to dichlorodiphenyltrichloroethane (DDT), permethrin, deltamethrin, lambda-cyhalothrin, etc (Jangir and Prasad, 2022; Sumitha et al., 2023). In many areas, insecticide resistance is becoming increasingly common, necessitating the development of innovative, sustainable, and ecologically friendly methods (Qadri et al., 2020; Vlaiculescu and Varrone, 2022; Washim et al., 2024). Novel approaches have been investigated, including the manipulation of mosquito symbionts, specifically the bacterium *Wolbachia pipientis* (Branda et al., 2024; Laven, 1967; Stouthamer et al., 1999).

*Wolbachia pipientis* is a species of bacteria that is intracellular, maternally inherited, and infects a wide range of arthropods and nematodes. It was first characterized almost a century ago in *Culex pipiens* mosquitoes (Fallon, 2021; Minwuyelet et al., 2023). *Wolbachia* presents a promising substitute because it can both suppress mosquito populations through cytoplasmic incompatibility (CI) and prevent pathogen transmission through viral inhibition (Caragata et al., 2019a,b; Hu et al., 2020; Iturbe-Ormaetxe et al., 2011; Sarwar et al., 2022; Yen and Failloux, 2020). The ability of *Wolbachia* to control host reproduction is remarkable which involves a reproductive incompatibility between males infected with *Wolbachia* and uninfected females leading to either reduced offspring viability or total embryonic lethality. Although *Wolbachia* is present in many mosquito species, it is prominently absent from those that are thought to be the main carriers of human pathogens. Some studies report a prevalence of 28%–30% for natural *Wolbachia* infection in mosquitoes (Minwuyelet et al., 2023; Dorigatti et al., 2018; Kittayapong et al., 2000).

Many countries, including Australia, Indonesia, China, and Brazil, have reported successful field trials showing notable decreases in dengue incidence using *Wolbachia*-infected *Ae. aegypti* (Hu et al., 2020; Sarwar et al., 2022; Flores and O’Neill, 2018; Pinto et al., 2021). Such population replacement strategies rely on the assumption that local mosquito populations lack natural *Wolbachia* infections, highlighting the importance of establishing baseline data on native *Wolbachia* prevalence before any biocontrol programme can be considered. In this study, we aimed to detect and genetically characterize *Wolbachia* endosymbionts in *Ae. aegypti* populations from a recent arboviral outbreak region in Pune District, Maharashtra. Generating this baseline evidence is crucial for understanding the natural diversity of circulating *Wolbachia* strains and for assessing whether future *Wolbachia*-based biocontrol strategies could be feasible or require adaptation to local ecological conditions.

## 2 Methods

### 2.1 Study area

The entomological survey was conducted across 21 locations in Pune District, Maharashtra, India. The majority of selected sites were located within Pune city taluk, which reported the highest number of arboviral cases during the 2024 outbreak, according to official data from the Maharashtra State Health Department. Site selection was therefore prioritized based on reported case density and entomological feasibility to capture hotspots of arbovirus transmission. While the focus on Pune city enabled intensive sampling in outbreak-affected areas, we acknowledge that fewer sites were included from other talukas. This selection bias was a logistical constraint during the outbreak response phase, and future studies will aim to include broader spatial representation across the district for more generalizable findings.

The surveyed locations included urban, peri-urban, and rural settings. Urban areas comprised Bodpodi, Khadaki, Bhekrai Nagar, SBH Yerawada, Landae Wadi Bhosari, Thergaon, Vishrantwadi, Vaiduwadi Shivajinagar, Sus Goan, Punawal, Dhankawadi, Malwadi, and Maan. Rural sites included Theur, Kunjirwadi, Nere, Boore Budruk, and Morgaon. Peri-urban areas, characterized by transitional landscapes and mixed land use, included Lohegaon, Loni Kalbhor, and Takave. Although Takave is administratively classified as rural, it exhibits peri-urban characteristics due to ongoing urban expansion and infrastructure development.

**Figure 1:**
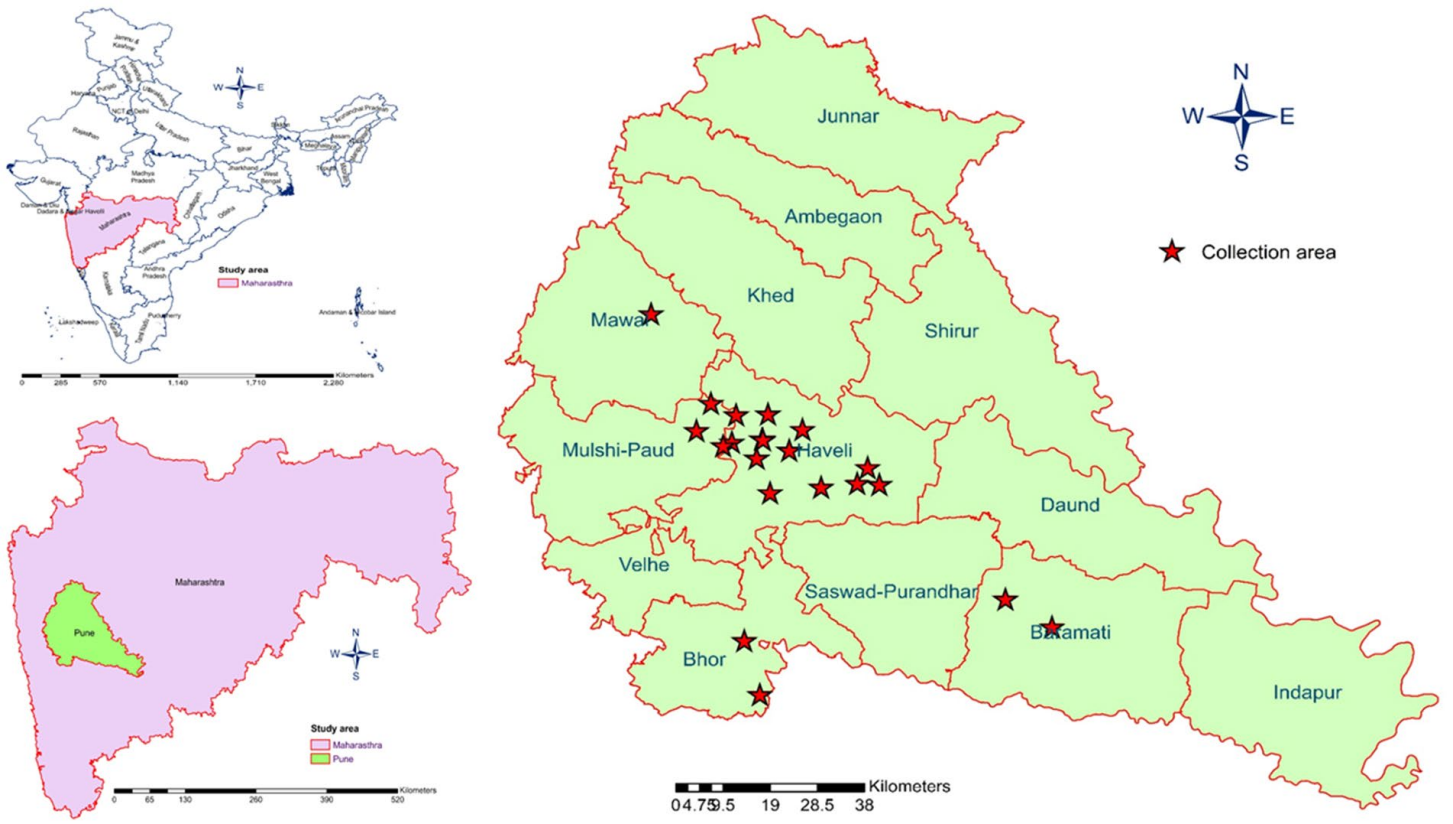
Map of Pune district showing the 21 selected locations surveyed for *Aedes* mosquito collection.

This classification facilitated stratified sampling and spatial analysis of entomological indices and arbovirus detection across ecologically distinct zones. The geographical distribution of the study sites is depicted in Figure 1.

### 2.2 Mosquito collection and identification

Immature and adult *Aedes* mosquitoes were collected from 21 locations in Pune District, Maharashtra, which were affected by a confirmed dengue and chikungunya outbreak in mid-2024. Entomological surveillance and mosquito sampling were conducted during the active transmission period from August to November 2024 (08:00–12:00 h), following notification from the Health Department of Maharashtra. This post-monsoon period is also known to coincide with peak *Aedes*-borne virus activity in the region. A total of 612 households were randomly surveyed using standardized entomological protocols (Padonou et al., 2023; WHO, 2016; Kumar et al., 2021; Kumar et al., 2021). Adult mosquitoes were collected from indoor and outdoor environments using oral and mechanical aspirators. Simultaneously, a larval and pupal survey was conducted by inspecting all potential water-holding containers located inside and around the surveyed households (Balaji et al., 2019; Shah and Sani, 2011; NCVBDC, 2022; NCVBDC, 2020).

Containers were considered positive if they exhibited the presence of at least one larva or pupa. A household was recorded as positive if it contained at least one such container. Larvae and pupae were collected from positive containers using a dipper or pipette and transferred into labeled 250 mL plastic containers. All samples were subsequently transported to the laboratory for further processing. To evaluate the density and spatial distribution of *Aedes* breeding, entomological indices were calculated for each sampling location. These included the Container Index (CI), House Index (HI), Breteau Index (BI), and Free Larva Index (FLI), following standard WHO and NCVBDC guidelines (WHO, 2016; NCVBDC, 2022; NCVBDC, 2020; Garjito et al., 2020). In the laboratory, all mosquito specimens were morphologically identified to species level using standard taxonomic keys. Adult specimens were further sorted by species and sex, fed, unfed gravid etc. For molecular analyses, mosquitoes were pooled in groups of up to 20 individuals and stored in sterile 2 mL Eppendorf tubes. Larval and pupal samples were also pooled by collection location and stored at −80 °C (Bikangui et al., 2023; Padonou et al., 2023; Kumar et al., 2021; Balaji et al., 2019; Garjito et al., 2020; Kumar et al., 2012; Badolo et al., 2022; Sanchez et al., 2006).

### 2.3 DNA extraction

The pooled samples were homogenized in Minimum Essential Medium supplemented with Fetal Bovine Serum. Then the cellular debris was separated using a centrifuge at 8000 rpm for 5 min, and the supernatant was used for DNA isolation. Extraction of genomic DNA was performed according to the manufacturer’s recommendations using a QIAamp DNA Mini Kit (Qiagen). The concentration and quality of isolated DNA was tested by absorbance at 260/280 using a nanodrop spectrophotometer (Chao and Shih, 2023; Hu et al., 2020; Somia et al., 2023; Zhang et al., 2022).

### 2.4 Molecular identification of mosquito species

The Morphologically identified *Ae. aegypti* samples are further confirmed by amplifying Cytochrome Oxidase I (COI), a mitochondrial gene. The PCR was performed with a total reaction volume of 50 μL comprising the following components: 25 μL of Takara Emerald Green master mix, 15 μL of d. dH2O, 2.5 μL of Forward primer, and 2.5 μL of Reverse primer, with 5 μL of template. The thermal cycle condition for the PCR is as follows: 95 °C for 5 min, 1 cycle Pre-cycle Denaturation step followed by 35 cycles of 94 °C for 30 s, 57 °C for 30 s, and 72 °C for 45 s with a final extension of 72 °C for 10 min. Species identification was achieved by sequencing the amplicon (Chao and Shih, 2023).

### 2.5 Detection of DENV, CHIKV and ZIKV in mosquito pools

The viral RNA was extracted from 140 μL suspension of mosquito pools using a commercial viral RNA extraction kit. The viral RNA was subjected to a two tube real-time RT-PCR assay for detection of DENV serotypes (DENV serotypes 1–4) and CHIKV as described earlier (Alagarasu et al., 2023a,b). ZIKV was detected using a real-time RT-PCR as reported previously (Lanciotti et al., 2008).

### 2.6 Detection of *Wolbachia*

A polymerase chain reaction (PCR) assay for amplification of the 16S rRNA gene was used for *Wolbachia* screening. The amplification was performed with *Wolbachia*-specific primers 16S WspecF (5′-CATACCTATTCGAAGGGATAG-3′) and 16S Wspec (5′- AGCTTCGAGTGAAACCAATTC-3′) as forward and reverse primers, respectively (Hu et al., 2020; Braig et al., 1998; Braig et al., 1998) which produce an amplicon spanning 438 bp within the 16S rRNA region. The PCR was performed with a total reaction volume of 10 μL comprising the following components: 5 μL of Takara Emerald Green master mix, 3 μL of dH2O, 0.5 μL of Forward primer, and 0.5 μL of Reverse primer, with 1 μL of template. The thermal cycle conditions for the PCR are as follows: 95 °C for 5 min, followed by 1 cycle of pre-cycle denaturation, and then 35 cycles of 94 °C for 30 s, 55 °C for 30 s, and 72 °C for 45 s, with a final extension at 72 °C for 10 min. 5 μL of the product was loaded into a 1.5% agarose gel and resolved by electrophoresis (Misailidis et al., 2024; Muharromah et al., 2023).

### 2.7 Strain detection

Three different *wsp* (*Wolbachia* surface protein) gene primers have been used to detect super-group A and super-group B. For super-group A, primers used: 328F: (5′-CCAGCAGATACTATTGCG-3′) and 691R: (5′-AAAAATTAAACGCTACTCCA-3′) and for Super-group B, two different primers were used (i) 81F: (TGGTCCAATAAGTGATGAAGAAAC); 522R: (ACCAGCTTTTGCTTGATA) & (ii) 183F: (AAGGAACCGAAGTTCATG); 691R: (AAAAATTAAACGCTACTCCA) (Chao and Shih, 2023; Zhang et al., 2022; Braig et al., 1998; Carvajal et al., 2019).

The PCR was performed with a total reaction volume of 50 μL comprising the following components: 25 μL of Takara Emerald Green master mix, 15 μL of d. dH2O, 2.5 μL of Forward primer, and 2.5 μL of Reverse primer, with 5 μL of template. The thermal cycle conditions for super-group A and 183F;691R are as follows: 95 °C for 5 min, followed by 1 cycle of pre-cycle denaturation, and then 35 cycles of 94 °C for 30 s, 55 °C for 30 s, and 72 °C for 45 s, with a final extension at 72 °C for 10 min. For the primers 81F;522R, the PCR mixture compositions are the same, and the thermal conditions for the primer as follows: 95 °C for 5 min, followed by 1 cycle of pre-cycle denaturation, and then 35 cycles of 94 °C for 30 s, 58 °C for 30 s, and 72 °C for 45 s, with a final extension at 72 °C for 10 min. The primers of Supergroup A are expected to amplify a product of approximately 380 base pairs, and the primers of Supergroup B are expected to produce an amplicon of approximately 501 bp and 442 bp. 5 μL of the PCR products with 100 bp ladder was loaded into a 1.5% agarose gel and resolved by electrophoresis (Kittayapong et al., 2000; Hu et al., 2020; Somia et al., 2023; Zhang et al., 2022; Misailidis et al., 2024; Muharromah et al., 2023; Muharromah et al., 2023; Carvajal et al., 2019; Nugapola et al., 2017).

### 2.8 Sequencing and phylogenetic analysis

#### 2.8.1 Phylogenetic analysis of *Ae. Aegypti* COI gene sequences

To confirm the species identity and assess the phylogenetic placement of field-collected mosquitoes, one representative PCR-amplified sequences of the mitochondrial cytochrome c oxidase subunit I (COI) gene from morphologically identified *Ae. aegypti* specimens were selected. These sequences were compared with 50 closely related sequences retrieved from NCBI GenBank using BLASTn. Multiple sequence alignment was performed using MAFFT v7.490, followed by automatic trimming using trimAl v1.4.rev15, as well as manual curation to remove gaps and ambiguous regions. A neighbor-joining (NJ) phylogenetic tree was constructed in SeaView v4 using default parameters, and node support was assessed with 1,000 bootstrap replicates. The tree was rooted using sequences from *Aedes* japonicus and *Culex gelidus* as outgroup taxa. Tree visualisation was performed using FigTree version 1.4.4.

#### 2.8.2 Phylogenetic analysis of *Wolbachia wsp* gene sequences

PCR products displaying clear, single amplicon bands were directly subjected to Sanger sequencing via capillary electrophoresis. Samples showing nonspecific amplification were first purified using the QIAquick PCR & Gel Cleanup Kit (Qiagen, Germany) before sequencing. The resulting chromatograms were quality-checked, and nucleotide sequences were analyzed using BLASTn against the NCBI GenBank database to confirm gene identity. Sequence similarity was determined based on the top BLAST hits retrieved. For phylogenetic analysis, the *Wolbachia* surface protein gene (wsp) was sequenced from 9 *Wolbachia*-positive *Ae. aegypti* isolates. Each sequence was individually analyzed using BLASTn to identify the top four most similar reference sequences available in GenBank. These sequences, along with the corresponding field isolate sequences, were aligned using MAFFT v7.490. The alignments were generated using automatic trimming with trimAl v1.4.rev15, as well as manual curation to remove gaps and ambiguous regions. This process involved manually curated removal of poorly aligned regions, gaps, and ambiguous bases. Phylogenetic relationships were inferred using the neighbor-joining (NJ) implemented in SeaView v4. Branch support was assessed using 1,000 bootstrap replicates. The final NJ phylogenetic trees were visualized using FigTree v1.4.4.

### 2.9 Statistical analysis

**Figure 2:**
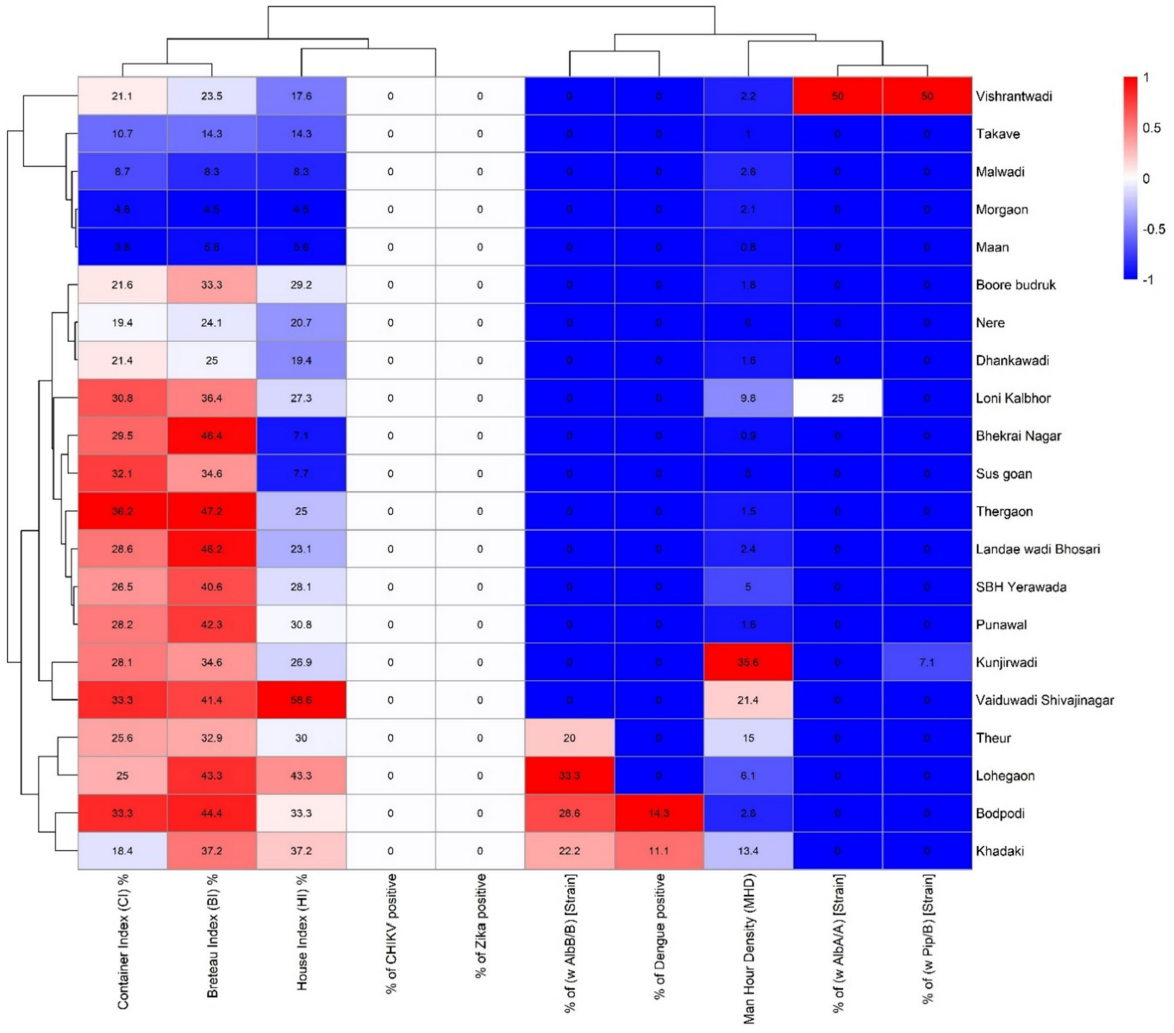
Heatmap showing entomological indices, arboviral positivity, and *Wolbachia* strain prevalence across 21 study locations in Pune district, India. The figure illustrates the spatial variation in key entomological indices container index (CI), Breteau index (BI), and house index (HI) alongside arbovirus positivity rates (% CHIKV, ZIKV, and DENV) and *Wolbachia* supergroup prevalence (*wAlbB*, *wAlbA*, and *wPip*) across 21 surveyed sites. The intensity of red indicates higher values, while blue represents lower or absent values. Hierarchical clustering was applied to group locations based on similar entomological and molecular characteristics. Notably, sites such as Vishrantwadi, Lohegaon, and Vaiduwadi Shivajinagar exhibited both high larval indices and *Wolbachia-*positive pools, along with arbovirus detections.

Data were analysed at the cluster level (*N* = 21) using R software (v4.5.1) with packages including tidyverse, pheatmap, and RColorBrewer. Descriptive statistics were used to summarise entomological indices (HI, CI, BI, MHD), *Wolbachia* strain prevalence (wAlbA, wAlbB, wPip), and arboviral RNA positivity (DENV, CHIKV, ZIKV). Means and 95% confidence intervals were calculated for continuous variables, while proportions were used for categorical data. Pearson’s correlation coefficients assessed associations between vector indices, Wolbachia prevalence, and arbovirus detection, with significance set at *p* < 0.05. A clustered heatmap (Figure 2) was generated using the pheatmap package. Variables were z-score standardised, and hierarchical clustering (Euclidean distance, Ward’s method) was applied to rows (clusters) and columns (indicators). The heatmap colour gradient (blue to red) visualised patterns of variation in entomological risk, endosymbiont presence, and arboviral activity across sites.

## 3 Results

### 3.1 Entomological surveillance indicators and vector density

**Figure 3:**
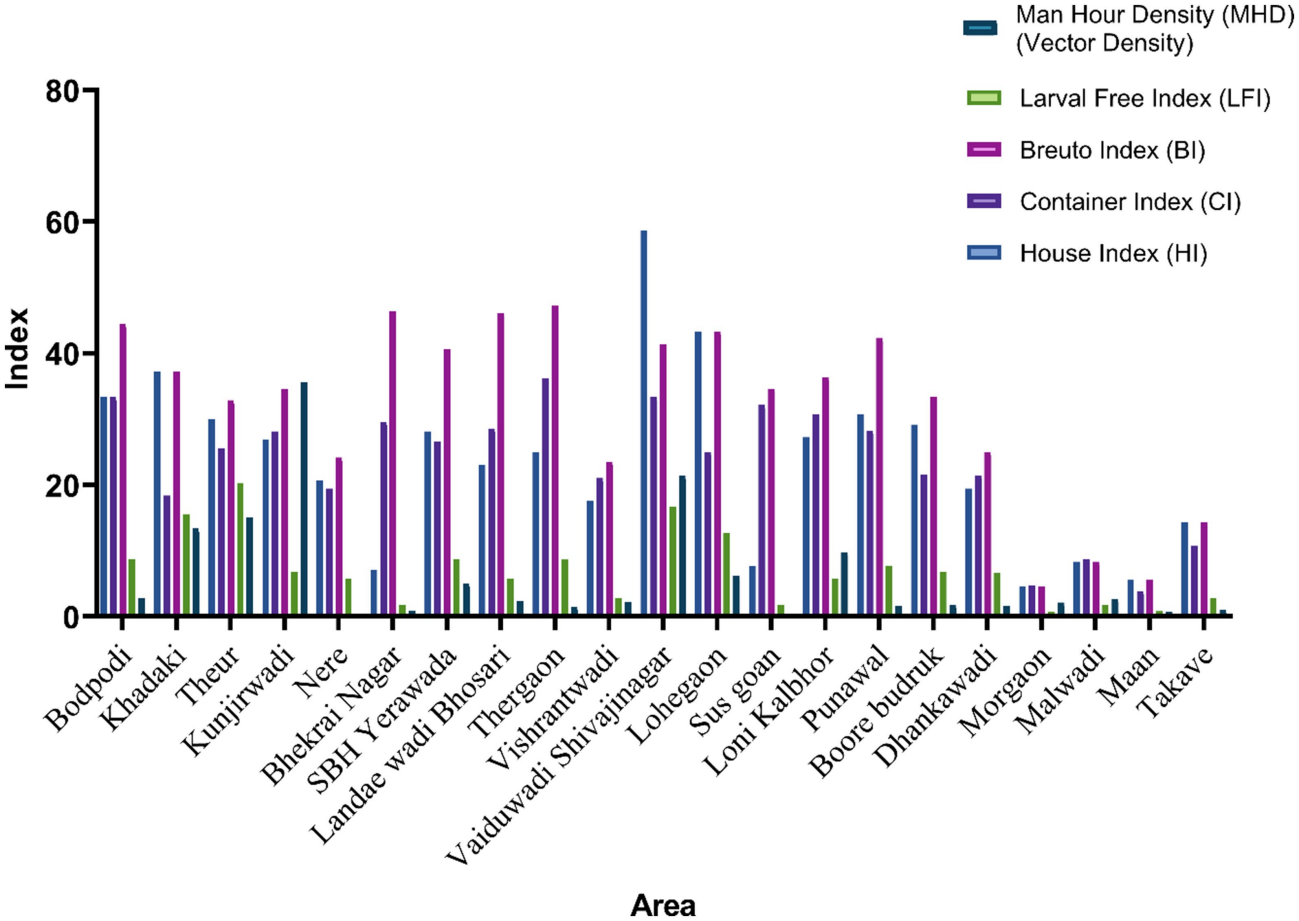
Comparison of entomological indices across 21 localities in Pune district, India. The figure displays the distribution of key vector surveillance indices for *Aedes aegypti* house index (HI), container index (CI), Breteau index (BI), larval free index (LFI), and man hour density (MHD) across 21 surveyed areas. The indices provide insights into larval infestation levels, adult mosquito density, and overall vector breeding risk. Higher BI and MHD values in several localities indicate zones with elevated transmission potential requiring targeted vector control interventions.

Comprehensive entomological investigations were conducted across 21 outbreak-affected localities in Pune district to assess *Aedes aegypti* larval infestation and adult mosquito density. Standard WHO-recommended indices House Index (HI), Container Index (CI), and Breteau Index (BI) were computed, along with the Larval Free Index (LFI) and Man Hour Density (MHD) to estimate adult vector abundance (Figure 3). A total of 612 households were surveyed, with an average of 29.1 houses per cluster (95% CI: 24.1–34.2). Of these, 7.4 houses per cluster (95% CI: 4.9–9.9) were positive for *Aedes* breeding, resulting in a mean House Index (HI) of 23.7% (95% CI: 17.6–29.9). The number of inspected water-holding containers per cluster averaged 40.0 (95% CI: 31.5–48.5), with 9.7 containers (95% CI: 7.1–12.2) found positive for larvae or pupae, yielding a mean Container Index (CI) of 23.2% (95% CI: 18.9–27.5) and a mean Breteau Index (BI) of 31.7% (95% CI: 25.5–38.0).

At the site level, HI varied markedly across study areas, ranging from 4.55% in Morgaon (rural) to 58.62% in Vaiduwadi Shivajinagar (urban), with 10 localities exceeding the WHO threshold of 10%, indicating active transmission risk. Similarly, CI ranged between 3.85% (Maan) and 33.33% (Bodpodi and Vaiduwadi Shivajinagar). BI was highest in Thergaon (47.22%), Bhekrai Nagar (46.43%), and Landae Wadi Bhosari (46.15%), all urban areas exceeding the 20% risk threshold. Comparative analysis revealed that urban and peri-urban clusters demonstrated consistently higher larval indices than rural sites. However, rural sites such as Kunjirwadi and Theur also exhibited high values, indicating active breeding despite lower container density. The Larval Free Index (LFI) was lowest in Bhekrai Nagar (1.72%), Sus Goan (1.74%), and Morgaon (0.78%), suggesting widespread infestation. Only Theur (LFI = 20.3%), Vaiduwadi Shivajinagar (16.7%), and Khadaki (15.6%) recorded Larval Free Index (LFI) values exceeding the 10% threshold. Since LFI reflects the percentage of inspected households with no larval presence, higher values indicate more effective control of larval habitats and reduced breeding potential of *Aedes* mosquitoes.

Adult mosquito surveillance yielded a mean Man Hour Density (MHD) of 6.1 (95% CI: 2.1–10.1) across sites. On average, 48.6 adult *Ae. aegypti* mosquitoes per cluster were collected (95% CI: 16.5–80.7), comprising similar numbers of males (24.9; 95% CI: 5.5–44.2) and females (23.7; 95% CI: 10.5–36.9). Highest MHDs were observed in Kunjirwadi (35.63), Vaiduwadi Shivajinagar (21.38), and Theur (15.00), correlating with high larval indices and denoting sustained vector abundance. Molecular identification revealed that all adult mosquito pools (100%) tested positive for *Ae. aegypti* (95% CI: 83.9–100.0), with no other *Aedes* species detected, confirming mono-vector dominance in the outbreak zone. A detailed summary of household, container, pupal, and adult mosquito data including *Wolbachia* strain prevalence and arboviral screening results is presented in Table 1. Figure 3 displays the comparative entomological indices (HI, CI, BI, LFI, MHD) across all surveyed localities.

**Table 1:** Data characteristics of study clusters.

Variable	*N* = 21 clusters^a^
Number of houses	29.1 (24.1, 34.2)
Number of houses, positive for species	7.4 (4.9, 9.9)
House index (HI) %	23.7 (17.6, 29.9)
Number of containers	40.0 (31.5, 48.5)
Number of positive containers	9.7 (7.1, 12.2)
Container index (CI) %	23.2 (18.9, 27.5)
Breteau index (BI) %	31.7 (25.5, 38.0)
Number of Pupa	11.9 (0.0, 29.4)
Number of male (adults)	24.9 (5.5, 44.2)
Number of female (adults)	23.7 (10.5, 36.9)
Number of *Aedes* (Adults)	48.6 (16.5, 80.7)
Man hour density (MHD)	6.1 (2.1, 10.1)
Species	
*Aedes aegypti*	21 (100.0%, 83.9–100.0)
Number of pools	4.4 (2.9, 6.0)
% of (w AlbA/A) [Strain]	3.6 (0.0, 9.0)
% of (w Pip/B) [Strain]	2.7 (0.0, 7.7)
% of (w AlbB/B) [Strain]	5.0 (0.1, 9.8)
% of Dengue positive	1.2 (0.0, 3.0)
% of CHIKV positive	0.0 (0.0, 0.0)
% of Zika positive	0.0 (0.0, 0.0)

a Mean (95% CI) or Count [% (95% CI)].

### 3.2 Detection of DENV, CHIKV and ZIKV in mosquito pools

Arboviral screening of a total of 93 mosquito pools revealed dengue virus (DENV) RNA in two pools, representing 2.1% of all tested pools and 2 out of 21 study clusters 1.2% (95% CI: 0.0–3.0). Notably, one DENV-2 positive pool from Khadki (ENT 24/MSC 15-2) was also positive for *Wolbachia* strain *wAlb*B, suggesting possible co-occurrence of endosymbiont and virus within the same mosquito pool. The second DENV-2 positive pool (ENT 24/MSC 1, Bopodi) was *Wolbachia*-negative. No pools tested positive for chikungunya virus (CHIKV) or Zika virus (ZIKV) in the current screening. These findings are summarized in Tables 1, 2.

**Table 2:** Pools details of *Wolbachia* and dengue positive.

Sl. no	Pool ID	Location	Species	Male/Female/Larva/Pupa	Strain specific			+ve of Dengue/CHICKV/Zika
					A	B (*Pip*)	B (*wAlb*B)	-ve
1	ENT 24/ MSC 25–4	Lohegaon	*Ae. aegypti*	Male	-ve	-ve	+ve	-ve
2	ENT 24/ MSC 27–7	Loni Kalbhor	*Ae. aegypti*	Male	+ve	-ve	-ve	-ve
3	ENT 24/ MSC 23–3	Visheantwadi	*Ae. aegypti*	Male	+ve	+ve	-ve	-ve
4	ENT 24/ MSC 23–4	Visheantwadi	*Ae. aegypti*	Female	+ve	+ve	-ve	-ve
5	ENT 24/ MSC 16–10	Kunjirwadi	*Ae. aegypti*	Female	-ve	+ve	-ve	-ve
6	ENT 24/ MSC 14–9	Theur	*Ae. aegypti*	Female	-ve	-ve	+ve	-ve
7	ENT 24/ MSC 15–1	Khadki	*Ae. aegypti*	Male	-ve	-ve	+ve	-ve
8	ENT 24/ MSC 15–2	Khadki	*Ae. aegypti*	Male	-ve	-ve	+ve	DENV-2
9	ENT 24/ MSC 14–1	Theur	*Ae. aegypti*	Pupa	-ve	-ve	+ve	-ve
10	ENT 24/ MSC 2	Bopodi	*Ae. aegypti*	Male	-ve	-ve	+ve	-ve
11	ENT 24/ MSC 4	Bopodi	*Ae. aegypti*	Larvae	-ve	-ve	+ve	-ve
12	ENT 24/ MSC 1	Bopodi	*Ae. aegypti*	Adult	-ve	-ve	-ve	DENV-2

### 3.3 Detection and characterization of *Wolbachia* in field-Collected *Ae. Aegypti*

A total of 1,020 adult *Ae. aegypti* (522 males and 498 females), along with 750 larvae and 250 pupae, were collected from multiple sites and grouped into 93 pools according to developmental stage, sex, and collection locality. All mosquito pools were confirmed as *Ae. aegypti* through morphological examination and molecular identification based on cox1 gene amplification for one pool. Each pool was individually screened for the presence of *Wolbachia* using 16S rRNA gene-specific primers. *Wolbachia* was detected in 11.8% of mosquito pools (11 out of 93 pools tested), representing pooled samples grouped by mosquito sex, developmental stage, and location. The positive detections were distributed across developmental stages, including six male pools, three female pools, one larval pool, and one pupal pool, indicating the presence of *Wolbachia* across multiple life stages (Table 2).

To determine the genetic lineage, all *Wolbachia*-positive samples were subjected to supergroup-specific PCR assays. Among the 11 positive pools, two pools were identified as belonging to supergroup A, while the remaining nine pools were assigned to supergroup B. Further strain differentiation within supergroup B revealed that three pools were positive with wsp183F/wsp691R [B(*Pip*)] primers, and seven pools were positive with wsp81F/wsp522R [B(*wAlbB*)] primers. These findings demonstrate the coexistence of multiple *Wolbachia* supergroups within field populations of *Ae. aegypti* in Pune District, with a predominance of supergroup B, particularly the *wAlbB* strain. The detection of *Wolbachia* in both adult and immature stages supports the potential for vertical transmission and suggests natural maintenance of the endosymbiont within local mosquito populations.

Further analysis of *Wolbachia* prevalence revealed low but variable strain detection across clusters: *wAlbA* was present in 3.6% of pools (95% CI: 0.0–9.0), wPip/B in 2.7% (95% CI: 0.0–7.7), and wAlbB in 5.0% (95% CI: 0.1–9.8).

### 3.4 Correlation between different entomological indices

Correlation analyses revealed strong relationships among entomological indices. The House Index correlated moderately with the Container Index (*r* = 0.53, *p* < 0.05) and Breteau Index (*r* = 0.61, *p* < 0.01), while CI and BI were very strongly correlated (*r* = 0.92, *p* < 0.001). Man Hour Density correlated moderately with HI (*r* = 0.51, *p* < 0.05), suggesting that higher larval infestation was associated with increased adult mosquito abundance.

Among *Wolbachia* strains, *wAlbA* and *wPip*/B were highly correlated (*r* = 0.88, *p* < 0.001), indicating possible co-circulation patterns, while *wAlbB* showed a significant positive correlation with dengue positivity (*r* = 0.64, *p* < 0.01). Interestingly, *Wolbachia* infections did not correlate significantly with larval indices, suggesting their dynamics are not directly dependent on breeding site positivity (Table 3). Figure 2 illustrates a clustered heatmap depicting relationships among entomological indices, *Wolbachia* infection rates, and arboviral positivity across the 21 clusters. Distinct clustering patterns emerged among both clusters and variables, reflecting heterogeneity in vector infestation and infection profiles. Clusters such as Vishrantwadi, Lohegaon, Bodpodi, and Vaiduwadi Shivajinagar exhibited higher infestation levels, indicated by elevated CI, BI, and HI values (red tones), alongside detectable *wAlbB* strain and sporadic dengue positivity. Conversely, clusters including Takave, Malwadi, ceMorgaon, and Maan had low HI, CI, and BI values (blue tones), and absence of *Wolbachia* infection, and no arboviral markers. Intermediate clusters such as Thergaon, SBH Yerawada, and Landae Wadi Bhosari showed moderate entomological indices but low viral detection, indicating possible transitional transmission dynamics or partial infestation control.

**Table 3:** Pearson correlation coefficients with significance stars.

Variable	House index (HI) %	Container index (CI) %	Breteau index (BI) %	Man hour density (MHD)	% of (w AlbA/A) [Strain]	% of (w Pip/B) [Strain]	% of (w AlbB/B) [Strain]	% of Dengue positive
House index (HI) %	1	0.53*	0.61**	0.51*	−0.07	−0.09	0.47*	0.28
Container index (CI) %	0.53*	1	0.92***	0.25	0.04	−0.04	0.14	0.13
Breteau index (BI) %	0.61**	0.92***	1	0.21	−0.09	−0.13	0.3	0.23
Man hour density (MHD)	0.51*	0.25	0.21	1	−0.05	0.01	0.13	0.05
% of (w AlbA/A) [Strain]	−0.07	0.04	−0.09	−0.05	1	0.88***	−0.14	−0.1
% of (w Pip/B) [Strain]	−0.09	−0.04	−0.13	0.01	0.88***	1	−0.12	−0.08
% of (w AlbB/B) [Strain]	0.47*	0.14	0.3	0.13	−0.14	−0.12	1	0.64**
% of Dengue positive	0.28	0.13	0.23	0.05	−0.1	−0.08	0.64**	1

*p* < 0.05, **p < 0.01, ***p < 0.001.

Hierarchical clustering grouped HI, CI, and BI tightly, consistent with their biological association in larval and pupal infestation. These indices clustered closely with MHD, representing adult densities. *Wolbachia* strain proportions with weaker correlations to vector density metrics indicated that Wolbachia prevalence was not directly dependent on mosquito abundance at this stage. Overall, the heatmap highlights spatial heterogeneity in vector infestation and infection, underscoring high-risk clusters and viral transmission potential.

### 3.5 Phylogenetic analysis

#### 3.5.1 Phylogenetic analysis of *Ae. Aegypti* based on COI gene sequences

**Figure 4:**
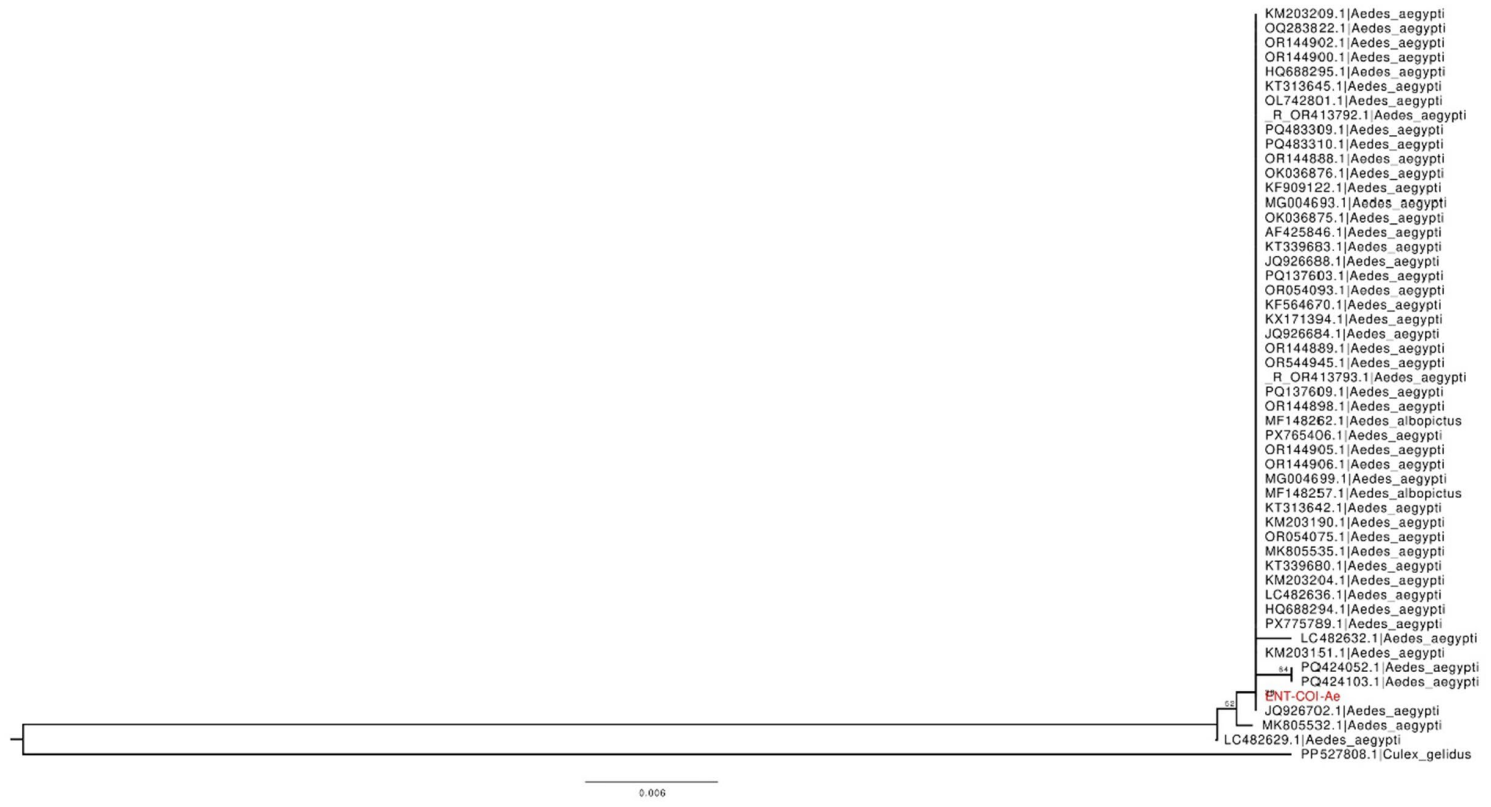
Neighbor-joining (NJ) phylogenetic tree based on partial cytochrome c oxidase subunit I (COI) gene sequences of *Ae. aegypti*. The tree includes the NIV-generated COI sequence (ENT-COI_Ae, shown in red) and representative *Ae. aegypti* reference sequences retrieved from GenBank. Phylogenetic analysis was performed in SeaView using default parameters, with node support assessed by 1,000 bootstrap replicates. *Culex gelidus* were included as outgroup taxa to root the tree. Bootstrap support values greater than 50% are shown at the nodes. The NIV COI sequence clusters unambiguously within the *Ae. aegypti* clade, confirming its species identity.

Species identity and evolutionary placement of field-collected *Ae. aegypti* specimens were confirmed by analyzing the cytochrome c oxidase subunit I (COI) gene sequence from the NIV isolate (ENT-COI-Ae) for this GenBank assesion No. PX909740 using a neighbor-joining (NJ) phylogenetic approach (Figure 4). The NJ tree was generated from a curated alignment of the top 50 COI sequences retrieved by BLAST against the NIV sequence, with *Culex gelidus* serving as an outgroup to root the phylogeny. The NIV COI sequence clustered clearly within the *Ae. aegypti* clade and grouped with reference sequences from diverse geographic regions, including Cameroon (JQ926702) and Colombia (KM203151) (Figure 4). The tree topology indicated limited geographic structuring, which aligns with the conserved nature of mitochondrial COI sequences in *Ae. aegypti*. No evidence of cryptic species or significant genetic divergence was detected, supporting the morphological and molecular identification of the NIV specimen as *Ae. aegypti*. These results demonstrate that, *Ae. aegypti* populations in Pune are genetically consistent with global *Ae. aegypti* populations at the mitochondrial level.

#### 3.5.2 Phylogenetic analysis of *Wolbachia wsp* gene sequences

**Figure 5:**
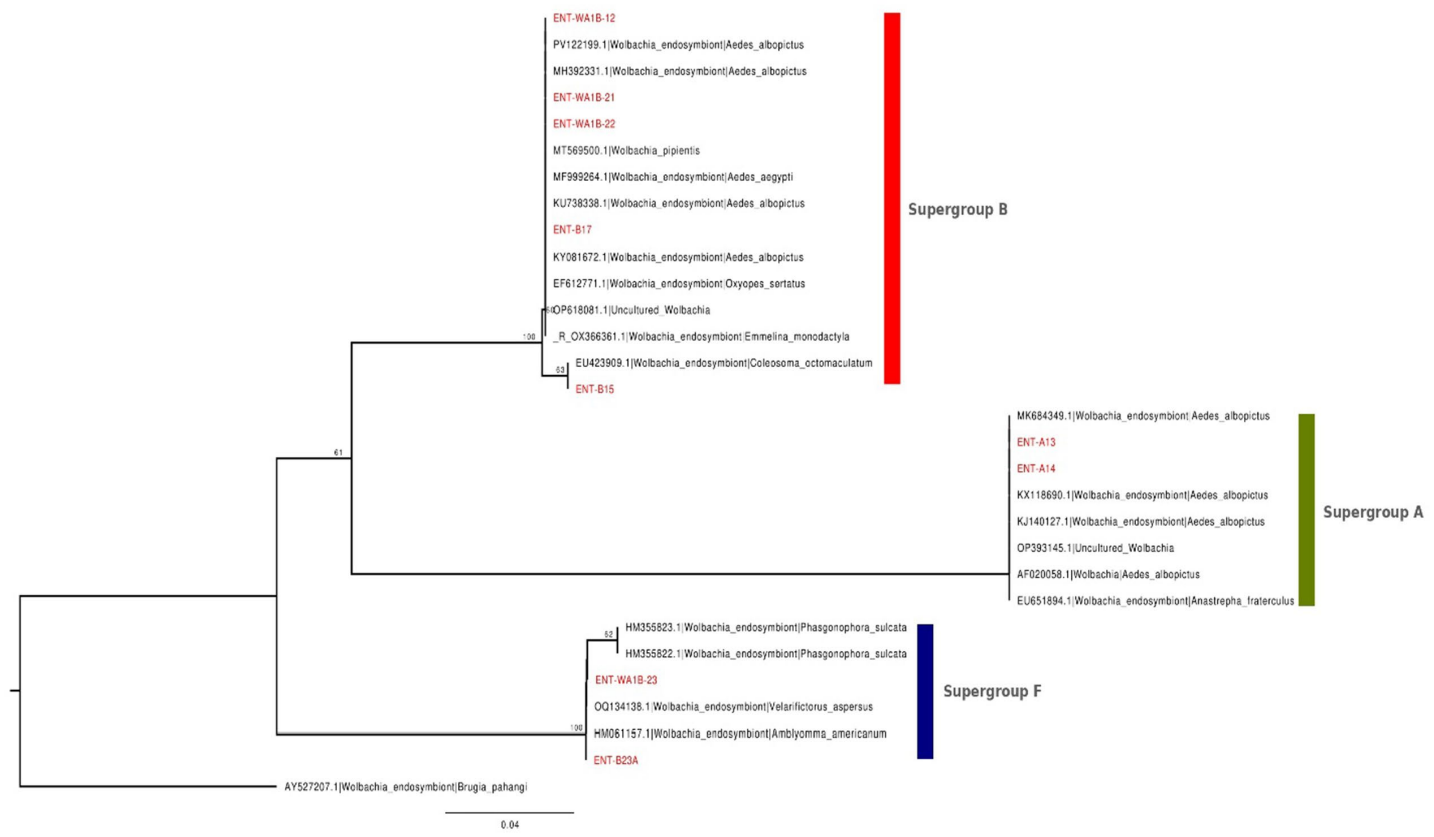
Neighbor-joining phylogenetic tree based on the *Wolbachia* surface protein gene (*wsp*) sequences. The tree includes nine sequences obtained from *Ae. aegypti* mosquitoes collected in Pune District (NIV strains, shown in red) and representative similar *wsp* sequences from *Ae. albopictus*, *Ae. aegypti*, and other arthropod hosts retrieved from GenBank. The analysis was performed using Seaview v4, with 1,000 bootstrap replicates. Branch labels indicate bootstrap percentages. The scale bar represents the number of substitutions per site.

Nine *Wolbachia wsp* gene sequences were obtained from *Ae. aegypti* mosquitoes collected in Pune, India, and analyzed to determine their phylogenetic relationships. A neighbor-joining (NJ) tree was constructed (Figure 5) using reference *wsp* sequences from GenBank, representing *Wolbachia* strains from various *Aedes* species and other arthropod hosts. The phylogenetic analysis showed that the NIV-derived sequences clustered into three distinct supergroups: A, B, and F. A member of supergroup D (AY527207.1) was used as the outgroup. Within supergroup A, NIV Within supergroup A, the NIV sequences ENT-A13 (PX912129) and ENT-A14 (PX912130) clustered with *Ae. albopictus*-associated reference strains (MK684349.1, KX118690.1, KJ140127.1, AF020058.1, OP393145.1, and EU561894.1).

Supergroup B comprised the majority of NIV-derived sequences, including ENT-WA1B-12 (PX912128), ENT-B15 (PX912131), ENT-B17 (PX912132), ENT-WA1B-21 (PX912133), and ENT-WA1B-22 (PX912134). These sequences grouped with *Wolbachia* reference strains associated with *Ae. albopictus*, *Ae. aegypti*, and other arthropod hosts, such as PV122199.1, MH392331.1, KU738338.1, KY081672.1, MT569500.1, MF999264.1, EU423909.1, and EF612771.1. The clustering was supported by moderate to high bootstrap values (>60), confirming their affiliation with the wAlbB lineage, which is widely reported in *Aedes* mosquitoes. Notably, two NIV-derived sequences, ENT-WA1B-23 (PX912135) and ENT-B23A (PX912136), grouped within Wolbachia supergroup F. These sequences formed a well-supported clade (bootstrap = 100) with *Wolbachia* reference sequences derived from non-mosquito arthropod hosts, including HM355822.1 and HM355823.1 (*Phasgonophora sulcata*), OQ134138.1 (*Velarifictorus aspersus*), and HM061157.1 (*Amblyomma americanum*). The placement of both Pune-derived sequences within supergroup F suggests divergence from the canonical *Aedes*-associated *Wolbachia* lineages (wAlbA and wAlbB). This pattern may reflect historical horizontal transmission events, environmental acquisition, or transient associations rather than stable vertical inheritance. However, as these observations are based solely on wsp gene sequences from pooled mosquito samples, further confirmation using multilocus sequence typing (MLST), quantitative PCR, and intracellular localization assays will be required to determine whether these sequences represent genuine *Wolbachia* infections in *Ae. aegypti*.

### 3.6 Intergenomic similarity analysis of *Wolbachia wsp* sequences

**Figure 6:**
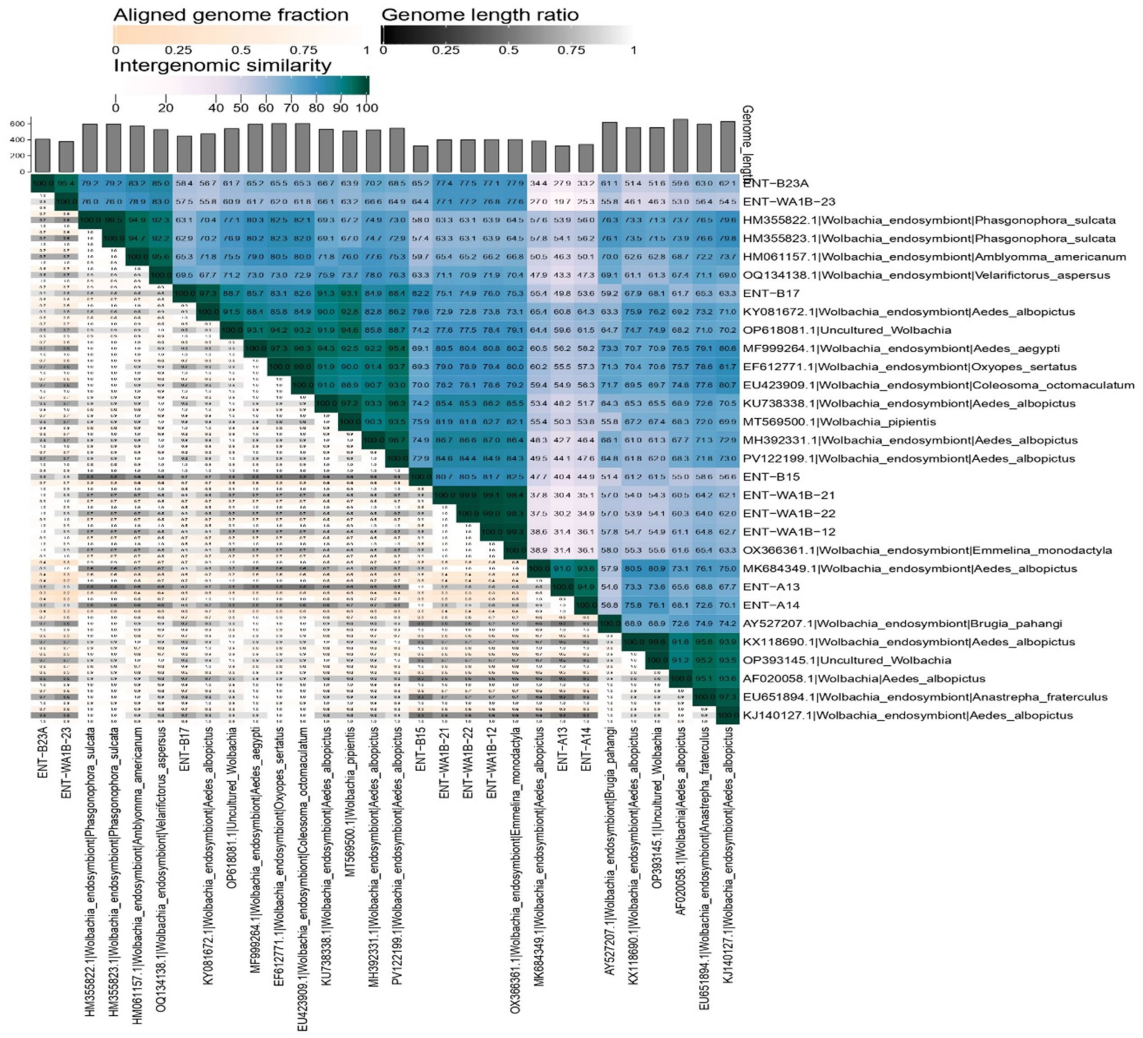
Intergenomic similarity heatmap and pairwise comparison matrix of *Wolbachia wsp* sequences. The matrix shows pairwise identity (%) between each sequence pair, with color gradients representing degrees of similarity. The aligned genome fraction and genome length ratio are displayed above the diagonal. NIV sequences from this study are highlighted on both axes. Closely related strains cluster in darker blue (≥80%), while lower similarity values (white to light blue) suggest divergent or uncharacterized lineages.

To further investigate the genomic relationships among *Wolbachia* strains, a pairwise intergenomic similarity matrix was generated based on aligned sequence identities values among 30 *wsp* gene sequences, including nine NIV-derived sequences and reference sequences from *Ae. albopictus*, *Ae. aegypti*, and other arthropod hosts. The resulted clustering heatmap (Figure 6) using VIRIDIC revealed three distinct genomic clusters that correspond closely with the supergroups identified by our NJ phylogeny (Figure 5).

Sequences within supergroup A (ENT-A13 and ENT-A14) shared high intergenomic similarity with each other, but exhibited low similarity (~20%–35%) to sequences belonging to supergroups B and F, consistent with their placement on a distinct phylogenetic branch. Likewise, supergroup B sequences (ENT-B15, ENT-B17, ENT-WA1B-12, ENT-WA1B-21, and ENT-WA1B-22) displayed moderate to high similarity among themselves, while showing low intergenomic similarity (<30–40%) when compared with supergroup A and F members, supporting their monophyletic clustering with the wAlbB lineage. Members of supergroup F formed a separate genomic cluster, characterized by higher within-group similarity but consistently low similarity (<30%) to both supergroup A and B sequences. Overall, inter-supergroup comparisons (A–B, A–F, and B–F) showed marked genomic divergence, directly reflecting the major phylogenetic splits in the wsp tree and confirming that genome-wide similarity patterns reinforce the inferred evolutionary relationships among *Wolbachia* supergroups. The VIRIDIC similarity matrix quantitatively confirms the three major supergroups (A, B, and F) identified by phylogenetic analysis. The strong agreement between similarity-based clustering and NJ topology supports the conclusion that *Ae. aegypti* populations in Pune contain a mix of *wAlbA*-related, *wAlbB*-derived, and highly divergent F-type *Wolbachia*, indicating substantial endosymbiont diversity and complex evolutionary interactions in Indian mosquito populations.

## 4 Discussion

This study presents novel insights into the natural occurrence, genetic diversity, and phylogenetic relationships of *Wolbachia* endosymbionts in *Ae. aegypti* populations collected from a recent chikungunya outbreak region in Pune District, Maharashtra. The detection of *Wolbachia* in 11.8% of mosquito pools, with a predominance of supergroup B (notably *wAlbB* and *wPip*-like strains), aligns with emerging global evidence of a low but notable presence of natural *Wolbachia* infections in *Ae. aegypti* populations across India, China, Saudi Arabia, and Sri Lanka (Balaji et al., 2019; Hu et al., 2020; Somia et al., 2023; Zhang et al., 2022; Nugapola et al., 2017).

Historically, *Ae. aegypti* was believed to lack natural *Wolbachia* infections (Kittayapong et al., 2000; Iturbe-Ormaetxe et al., 2011), a belief that underpinned early *Wolbachia*-based vector control strategies using artificially transinfected mosquitoes. However, recent findings including those reported in this study challenge this assumption. In India, Balaji et al. (2019) observed a 5.6% prevalence in field-collected *Ae. aegypti* from Tamil Nadu, while Chao and Shih (2023) reported 12.9% prevalence in southern Taiwan, comparable to our findings (Balaji et al., 2019; Chao and Shih, 2023). The detection of both *wAlbA* and *wAlbB* supergroups in this study indicates the co-circulation of multiple *Wolbachia* strains, a phenomenon similarly observed in *Ae. albopictus* populations in China (Hu et al., 2020; Misailidis et al., 2024) and *Ae. aegypti* in Jeddah (Somia et al., 2023). These patterns suggest possible horizontal transmission or introgression between sympatric mosquito species.

Strain-level identification revealed *wAlbB* as the most prevalent, supporting its ecological fitness and vertical transmission capacity, consistent with its persistence in *Ae. albopictus* and its use in population replacement programs (Pinto et al., 2021; Flores and O’Neill, 2018). The unexpected detection of *wPip*-like sequences typically associated with *Culex pipiens* in *Ae. aegypti* may suggest rare inter-species transmission events in ecologically diverse urban habitats like Pune, where multiple mosquito species co-occur (Laven, 1967; Braig et al., 1998). Phylogenetic analysis based on the *wsp* gene, supported by intergenomic similarity (VIRIDIC) analysis, resolved the detected *Wolbachia* sequences into three distinct supergroups: A, B, and F. Supergroup B was the most prevalent, with sequences clustering closely with *wAlbB* reference strains commonly associated with *Ae. albopictus*. This finding aligns with reports suggesting that *wAlbB* represents one of the most ecologically successful *Wolbachia* lineages, characterized by efficient vertical transmission and persistence across host populations (Pinto et al., 2021; Flores and O’Neill, 2018). Although some sequences displayed moderate divergence from canonical *wAlbB*, their consistent placement within supergroup B across phylogenetic and similarity analyses suggests local strain variation rather than misclassification.

Supergroup A was detected at lower frequency, with sequences clustering with *wAlbA* reference strains from *Ae. albopictus* and other dipteran hosts. The co-occurrence of supergroups A and B within *Ae. aegypti* populations has been reported previously in *Ae. albopictus* and sporadically in *Ae. aegypti* from regions where both species coexist (Hu et al., 2020; Somia et al., 2023; Misailidis et al., 2024). These observations raise the possibility of historical horizontal transfer or introgression facilitated by shared ecological niches, although definitive mechanisms remain unresolved.

Of particular interest was the identification of a divergent sequence clustering within supergroup F, grouping with *Wolbachia* strains from non-dipteran hosts such as *Phasgonophora sulcata* and *Amblyomma americanum*. Intergenomic similarity analysis revealed relatively low nucleotide identity with known mosquito-associated *Wolbachia*, supporting its classification as an atypical lineage. While supergroup F has been primarily reported from arthropods outside Diptera, occasional detections in mosquitoes have been described (Carvajal et al., 2019; Nugapola et al., 2017). The presence of this lineage in *Ae. aegypti* may reflect an ancestral association, rare horizontal transmission, or an underexplored symbiotic lineage maintained at low prevalence.

The detection of *Wolbachia* in both immature (larvae and pupae) and adult stages supports vertical transmission, though likely at low density and with spatial heterogeneity. Similar trends have been observed in Sri Lanka and the Philippines, where *Wolbachia* infections were localized and strain-specific (Carvajal et al., 2019; Nugapola et al., 2017). Furthermore, a positive correlation between wAlbB prevalence and DENV detection was observed in our exploratory analysis; however, this was based on only two DENV-positive pools and should be interpreted with caution. The pooled nature of samples and limited number of positive outcomes preclude biological inference. Further individual-level studies are needed to clarify any potential interaction (Dorigatti et al., 2018; Yen and Failloux, 2020). However, further experimental studies are needed to clarify whether this association reflects antiviral effects, ecological overlap, or confounding vector traits, especially since *Wolbachia* strain effects on virus suppression vary (Caragata et al., 2019a; Caragata et al., 2019b).

Entomological indices namely, the House Index (HI), Container Index (CI), and Breteau Index (BI) were significantly correlated with adult mosquito densities (MHD), reinforcing their utility for transmission risk assessment (Shah and Sani, 2011; Sanchez et al., 2006). Clusters such as Vaiduwadi Shivajinagar and Kunjirwadi, which exhibited high larval indices, *Wolbachia* positivity, and dengue detection, represent potential hotspots for targeted interventions. The absence of a direct correlation between *Wolbachia* prevalence and larval indices suggests that symbiont dynamics may operate independently of breeding site density, as similarly reported in Greek *Ae. albopictus* populations (Misailidis et al., 2024).

The detection of genetically distinct and potentially novel *Wolbachia* strains in this outbreak-affected region adds to the growing evidence of complex symbiont dynamics within *Ae. aegypti* populations. These observations warrant broader genomic investigations to unravel the evolutionary relationships, host interactions, and functional roles of these strains. Furthermore, the presence of both canonical and atypical *Wolbachia* lineages highlights the importance of continued molecular surveillance to inform vector biology and control strategies. Integrating such data with entomological and virological findings will be crucial for guiding future interventions and improving the understanding of arbovirus transmission dynamics in endemic settings.

Our correlation analysis revealed a positive association between wAlbB strain prevalence and dengue virus detection; however, this observation is based on only two DENV-positive mosquito pools and should be interpreted with caution. The pooled nature of the mosquito samples limits the ability to determine individual co-infection status, and the small number of clusters (*n* = 21) increases the likelihood that correlations may be influenced by a few extreme values. While these associations may be biologically suggestive, we consider the analysis exploratory in nature. Additionally, given the number of comparisons made, the potential for chance findings is acknowledged. These limitations underscore the need for future individual-level analyses with larger sample sizes and more robust statistical corrections to validate any potential interactions between *Wolbachia* prevalence and arboviral infection status in field populations.

### 4.1 Future directions

The detection of three distinct *Wolbachia* supergroups wAlbA, wAlbB, and a putative supergroup F in *Aedes aegypti* populations from Pune reveals notable genetic diversity rarely reported in Indian mosquitoes. While wAlbA and wAlbB have been documented globally, their natural occurrence in *Ae. aegypti* in India is uncommon. The clustering of one sequence within supergroup F typically found in non-dipteran hosts raises the possibility of horizontal transmission or an underexplored endosymbiotic lineage.

Different *Wolbachia* strains have varying effects on vector competence. For example, wAlbB is linked to dengue virus suppression and has been used in successful field-based population replacement programs. However, the biological role of supergroup F remains unknown. Future laboratory studies will assess the virus-inhibitory potential of these strains, focusing on their effects on dengue and chikungunya virus replication, mosquito fitness, and vertical transmission. These investigations will support the development of locally tailored *Wolbachia*-based biocontrol strategies and advance understanding of *Wolbachia* mosquito virus interactions in Indian ecosystems.

## 5 Conclusion

This study presents the first comprehensive molecular and phylogenetic analysis of *Wolbachia* endosymbionts in *Ae. aegypti* populations collected from a arbovirus outbreak region in Pune, India. Our findings confirm the natural presence of *Wolbachia* in 11.8% (11/93) of mosquito pools from Pune District. The highest number of positive pools were detected in Bopodi (3), Khadki (2), Visheantwadi (2), and Theur (2). Most infections belonged to supergroup B, including wAlbB and wPip-like lineages, followed by wAlbA, which was detected in pools from Loni Kalbhor and Visheantwadi. Notably, a supergroup F strain was identified in a larval pool from Bopodi, marking a rare finding in Indian *Ae. aegypti* populations. These results highlight the genetic diversity and spatial heterogeneity of *Wolbachia* strains in the region, with implications for future biocontrol strategies. Phylogenetic reconstruction and intergenomic similarity analyses showed strong concordance, consistently resolving NIV-derived sequences into three distinct supergroups. The observed genetic divergence within supergroup B suggests the circulation of locally differentiated *Wolbachia* variants, while the detection of a supergroup F-associated strain points to a previously underexplored dimension of *Wolbachia* diversity in mosquitoes.

The detection of genetically diverse and potentially novel *Wolbachia* strains in *Ae. aegypti*, along with evidence of vertical transmission in both immature and adult stages, contributes to the growing recognition that natural infections may be more widespread than previously assumed. These results provide foundational data for exploring *Wolbachia*-based biocontrol strategies in India and emphasize the importance of incorporating molecular symbiont surveillance into arboviral vector monitoring and integrated vector management programs.

### 5.1 Limitations

This study has several limitations that warrant consideration. First, the cluster-level sample size (*n* = 21) is relatively small, potentially limiting the statistical power and generalizability of the findings. Second, mosquito specimens were screened in pools rather than individually, which restricts the ability to confirm co-infection status within single mosquitoes and may underestimate low-prevalence infections. While the minimum infection rate (MIR) was used to estimate *Wolbachia* and virus prevalence, this method provides a conservative estimate. A maximum likelihood estimation (MLE) approach would yield more robust prevalence estimates, particularly when the number of positive pools is low, and should be considered in future studies. Third, correlation analyses conducted between *Wolbachia* prevalence, entomological indices, and viral positivity were exploratory in nature. Given the small sample size and multiple comparisons without false discovery rate (FDR) correction, the observed associations may include chance findings and should be interpreted cautiously.

Finally, strain-level identification of *Wolbachia* was based on wsp gene analysis, which, while widely used, has recognized limitations due to recombination, hypervariability, and homoplasy. These issues can obscure true phylogenetic relationships and may result in misclassification or overestimation of strain diversity. Thus, the phylogenetic clustering results should be viewed as preliminary. Future work incorporating multilocus sequence typing (MLST) or whole-genome sequencing will be essential for more accurate and definitive characterization of Wolbachia strains and their evolutionary relationships.

## Data Availability

The nucleotide sequences generated in this study have been deposited in the NCBI GenBank database under the following accession numbers:PX912128 (ENT-WA1B-12), PX912129 (ENT-A13), PX912130 (ENT-A14), PX912131 (ENT-B15), PX912132 (ENT-B17), PX912133 (ENT-WA1B-21), PX912134 (ENT-WA1B-22), PX912135 (ENT-WA1B-23), PX912136 (ENT-B23A), and PX909740 (ENT-COI-Ae).
